# Effect of using the WeChat platform on the perioperative health education of parents of children who underwent transthoracic device closure of VSDs

**DOI:** 10.1186/s13019-020-01282-0

**Published:** 2020-09-15

**Authors:** Qi-Liang Zhang, Ning Xu, Shu-Ting Huang, Qiang Chen, Hua Cao

**Affiliations:** 1 Department of Cardiac Surgery, Fujian Maternity and Child Health Hospital, Affiliated Hospital of Fujian Medical University, Fuzhou, China; 2 Department of Cardiovascular Surgery, Union Hospital, Fujian Medical University, Fuzhou, China

**Keywords:** WeChat, Health education, VSD

## Abstract

**Objective:**

To explore the effects of using the WeChat platform on the perioperative health education of parents of children who underwent transthoracic device closure of ventricular septal defects (VSDs).

**Methods:**

Participants were divided into a WeChat group and a leaflet group. Responses to relevant questionnaires and clinical data were recorded and analyzed.

**Results:**

Before the operation, the scores of the Caretaker Knowledge Questionnaire in the WeChat group were significantly higher than those in the leaflet group. The scores of PSQ-18 in the WeChat group were significantly higher than those in the leaflet group. All the children in the WeChat group were followed up 1 month after discharge, while four children in the leaflet group were lost to follow-up. The rate of attrition in the leaflet group was significantly higher than that in the WeChat group. For the postoperative complications, there was no significant difference between the two groups.

**Conclusion:**

Perioperative health education for parents of children who undergo transthoracic device closure of VSDs through the WeChat platform can effectively enhance parents’ knowledge of care, improve parent satisfaction, which is an effective method to ensure convenient operation and reduce loss to follow-up.

## Introduction

Ventricular septal defects (VSDs) are among the most common congenital heart diseases, and surgical repair with cardiopulmonary bypass is the primary treatment [1, 2]. With the development of minimally invasive technology, transthoracic device closure of VSDs has been widely used in Chinese clinics with sound clinical effects [3, 4]. Such treatment has the advantages of minimal trauma, less bleeding, the avoidance of cardiopulmonary bypass, and the ability to be easily performed, so patients can recover quickly and get discharged 2–3 days after the operation [5]. Most patients with VSDs were admitted to the hospital on the day of or 1 day before the operation and discharged from the hospital 2–3 days after the operation. The patients’ parents performed the majority of perioperative preparation and postoperative recovery at home. Because of the lack of medical resources and expertise, many parents might be stressed or anxious about home care during the perioperative period [6–8]. Therefore, it is essential to support the parents’ practical medical information to obtain better home care results during the perioperative period [9]. Recently, many different social media technologies have been widely used in health education about chronic diseases [10]. However, there are few reports on using the WeChat platform to implement perioperative health education in cardiac surgery. Therefore, this study aimed to explore the effects of using the WeChat platform on the perioperative health education of parents of children who underwent transthoracic device closure of VSDs.

## Methods

### Calculation of the study sample size

Based on the pre-experiment and assuming the difference between the two independent populations was 10%, α = 0.05, and β = 0.2, the number of participants required in each group was set as 33. Assuming a 10% attrition rate, the total sample size required was 74 (37 per group).

### Research design

A prospective randomized controlled study was performed in this study. The relevant data of 74 patients who underwent transthoracic device closure of VSDs, were collected from June 2018 to June 2019. The inclusion criteria were as follows: 1. children with a restrictive VSD were suitable for device closure. 2. parents were the primary caregivers, and 3. parents had smartphones and could correctly use the WeChat platform. The exclusion criteria were as follows: 1. children who would undergo surgical repair of VSDs. 2. children with other congenital heart diseases and who required simultaneous surgical correction; 3. children with other organ diseases; and 4. those who refused to participate in the study or follow-up plan. The related clinical data were shown in Table 1.

**Table 1:** Demographic characteristics of patients and their parents in two groups

	WeChat group	Leaflet group	P
**Age (years)**	2.3 ± 1.8	2.5 ± 2.1	0.516
**Size of ventricular septal defect (mm)**	3.8 ± 0.9	3.6 ± 0.8	0.824
**Pulmonary pressure (mmHg)**	30.9 ± 12.4	33.2 ± 13.7	0.728
**Age of parents (years)**			
**< 25 or 25**	6	6	0.834
**26–30**	10	7	
**31–35**	13	14	
**36–40**	5	8	
**> 40 or 40**	3	2	
**Parents’ education level**			
**Under high school**	6	7	0.921
**High school**	12	14	
**Junior college**	13	11	
**Bachelor degree or higher**	6	5	
**Living condition**			
**Rural area**	25	23	0.626
**City**	12	14	

### Data acquisition

**Fig. 1:**
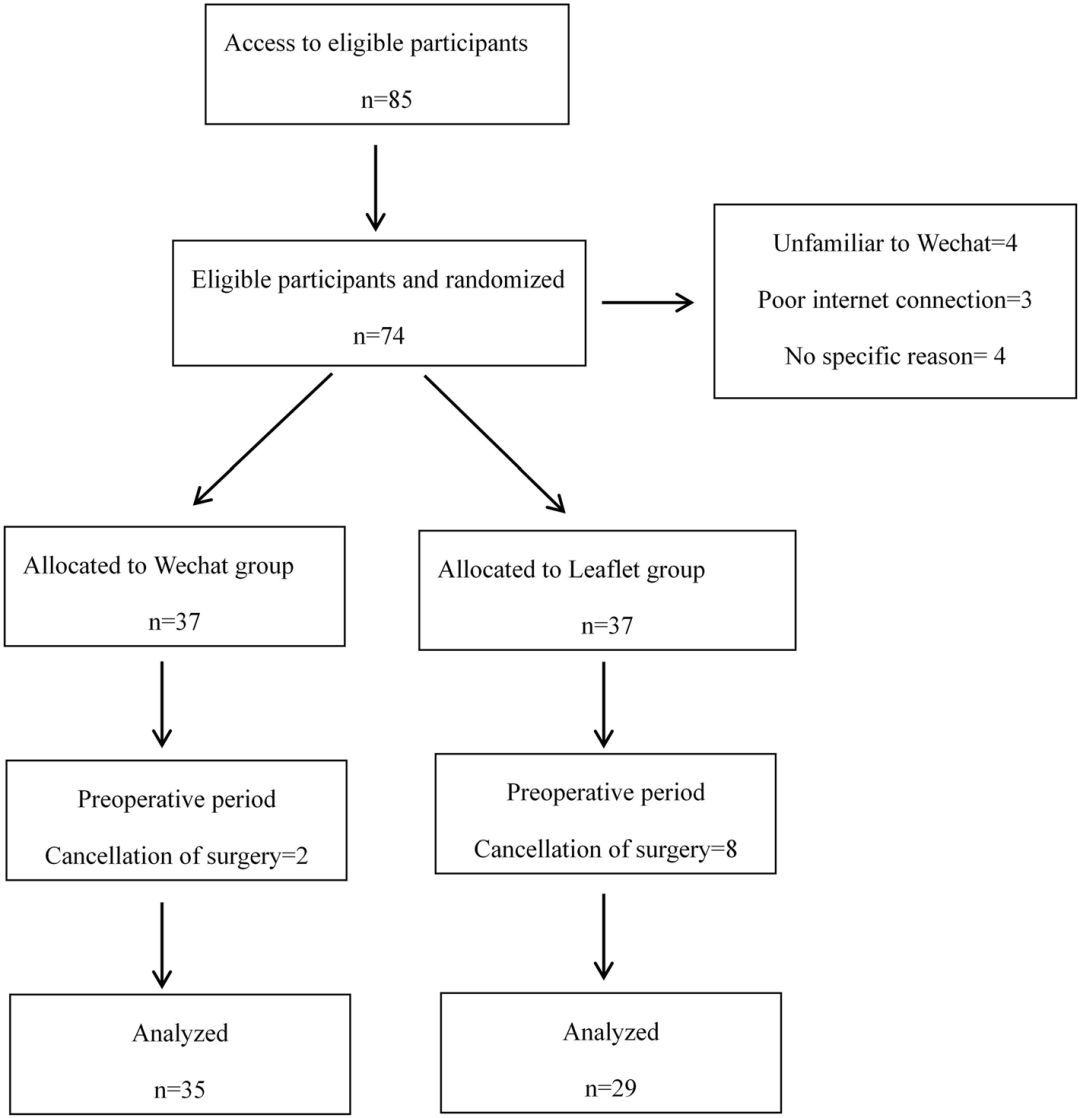
Flow chart

The eligible parents were randomly allocated to the intervention group (WeChat group) or the control group (leaflet group) based on random computer-generated numbers. The researcher reviewed the eligible parents for the study and collected the relevant data (Fig. 1). Parents were instructed not to disclose their group assignment or share any information with other parents.

### Intervention methods

Perioperative health education was provided for the parents in the intervention group via the WeChat platform. After determining the operation time, the children’s parents were guided to join the WeChat platform and helped to correctly and skillfully use the WeChat function. The content of health education in the WeChat platform was mainly included two parts: the education module and the answering and solving questions module. 1. The education module included knowledge about VSDs, preoperative care and feeding, and the management of related complications. Parents could view and learn these materials at any time of convenience. 2. In the answering and solving questions module, one medical staff member was online at 18:00–22:00 every day to explain information and reply to parents’ questions, remind parents about and supervise regular outpatient reviews, and remind parents of the precautions of the operative time and preoperative preparation. The medical staff also guided the family members to communicate, discuss, and share their care experiences and actively encourage each other in the WeChat group. If the medical staff found that some enthusiastic patients answer incorrectly, they would correct them in time.

The parents in the control group have distributed a leaflet on admission, which contained the same materials as the intervention group, the time of reexamination and operation, and some information about the disease. They were also instructed to visit the hospital if a sudden situation occurred.

### Research tool

This study focused on parents’ knowledge of the perioperative care of children with congenital heart disease, the rate of surgical cancellation, the rate of attrition 1 month after the operation, postoperative complications, and parents’ satisfaction with medical services. Surgical cancellation was defined as not undergoing surgery on the day of the planned operation.

The Caretaker Knowledge Questionnaire designed by professor Qiu was used to evaluate parents’ understanding of care for children with congenital heart disease [11]. The content validity, homogeneity reliability coefficient, and retest reliability of the questionnaire were 0.95, 0.84, and 0.82. The questionnaire contained 18 items, and each item is scored from 0 to 4 points. Except for items 11, 13, and 16, which were scored backward, they were scored forward. “Disagree” was 1 point; “toward disagree” was 2 points; “toward agree” was 3 points; “totally agree” was 4 points, and the total score was 72 points. A total score of < 44 was classified as a low knowledge level; 44 to 58 was classified as an intermediate knowledge level, and > 58 was classified as a high knowledge level. The higher the score was, the better the knowledge level.

The validated Patient Satisfaction Questionnaire-18 (PSQ-18 questionnaire) was used to assess parents’ satisfaction [12]. These eighteen questions of the questionnaire consisted of several sub-scales, including general satisfaction, technical quality, interpersonal manner, communication, financial aspects, time spent with the physician, accessibility, and convenience. The present questionnaire scores reflected the five-point Likert scale from 1 to 5.

### Statistical analysis

SPSS 18.0 software was used for statistical analysis. Continuous data were expressed as the mean ± standard deviation. A routine distribution test was performed for all continuous data, and the data were confirmed to have a normal distribution. Clinical parameters between the two groups were compared with the independent samples t-test. The χ2 or Fisher’s exact test was used for categorical variables. A *p*-value of < 0.05 was defined as statistically significant.

## Results

The scores of the Caretaker Knowledge Questionnaire in the WeChat group (68.8 ± 8.9) was significantly higher than those in the leaflet group (46.4 ± 10.5) in the perioperative period. (*P* = 0.010) The rate of cancellation of surgery in the WeChat group was significantly lower than that of the leaflet group (*p* = 0.041). All the children in the WeChat group followed up for 1 month after discharge, while four children in the leaflet group were lost to follow-up. The rate of attrition in the leaflet group (13.8%) was significantly higher than that in the WeChat group (*P* = 0.026). There was no significant difference between the two groups in the postoperative complications (such as pulmonary infections, arrhythmias, poor incision healing, residual shunts, and occluder detachment) (Table 2).

**Table 2:** Comparison of scores of Caretaker Knowledge Questionnaire, lost to follow-up and complications between the two groups

	WeChat group	Leaflet group	P
**Scores of Caretaker Knowledge Questionnaire**	68.8 ± 8.9	46.4 ± 10.5	0.010
**Cancellation of surgure**	2	8	0.041
**Lost-to-follow-up**	0	4	0.026
**Complications**			
**Pulmonary infections**	2	2	0.447
**Arrhythmias**	0	0	
**Poor incision healing**	0	0	
**residual shunts**	0	0	
**Occluder detachment**	0	0	

The results of the PSQ-18 showed that the scores of general satisfaction, technical quality, interpersonal manner, communication, time spent with a physician, and accessibility and convenience in the WeChat group were significantly higher than those in the leaflet group. There was no significant difference in technical quality and financial aspects between the two groups (Table 3).

**Table 3:** Comparison of scores of PSQ-18 between the two groups

	WeChat group	Leaflet group	P
**General satisfaction**	4.7 ± 0.8	3.2 ± 1.2	0.041
**Technical quality**	4.3 ± 0.9	4.2 ± 0.7	0.902
**Interpersonal manner**	4.6 ± 0.5	2.8 ± 0.9	0.033
**Communication**	4.8 ± 0.9	2.9 ± 1.0	0.027
**Financial aspects**	4.1 ± 0.7	4.2 ± 1.1	0.879
**Time spent with doctor**	4.9 ± 0.6	2.8 ± 1.3	0.013
**Accessibility and convenience**	4.9 ± 0.8	3.0 ± 1.1	0.020

## Discussion

VSD is one of the most common congenital heart diseases, and many children with VSD in China require surgical and interventional treatment [1–3]. For most patients with restricted VSD, transthoracic device closure of VSDs could be an option for many Chinese patients in the pre-school period. Advanced medical care is mainly concentrated in the large cities and a few medical centers, and these patients are usually hospitalized on the day of or 1 day before the operation and discharged from the hospital 2–3 days after the operation to save medical resources. Their parents mainly perform the patients’ preoperative preparation at home. However, due to the lack of medical knowledge and medical support, many patients’ perioperative preparation is imperfect. It is crucial to implement innovative strategies for patients and their parents for perioperative preparation. In recent years, mobile medical technology has been widely used as an educational tool for health care guidance [13–16]. WeChat can be used to spread disease-related information and provide health support by overcoming time and place constraints [17, 18]. In this study, a prospective randomized controlled study was performed to explore the effects of using the WeChat platform on perioperative health education for parents of children who underwent transthoracic device closure of VSDs.

This study showed that the rate of surgical cancellation in the leaflet group was significantly higher than in the WeChat group. The reason for surgical cancellation was mainly due to inadequate intestinal preparation and respiratory tract infection, which indicated that the parents could not adequately understand the knowledge necessary for preoperative preparation. Studies by Liu et al. and Kang et al. also showed that planned colonoscopies were canceled due to an incorrect gut preparation start time and incorrect dietary restrictions while moving multimedia applied to gut preparation education for colonoscopy improved the quality of colonoscopy gut preparation and reduced colonoscopy cancellation [19, 20]. Thus, it could be seen that full mastery of knowledge was an essential guarantee for disease control [21]. WeChat platform can expand health services and be an essential low-cost health education media. In our study, many parents in the control group were unable to master related knowledge from their leaflet because of professional restrictions and low education levels, even if they could appeal to their physician. Parents could learn about the disease, preoperative nursing, preoperative preparation, care and feeding, and the management of complications from the WeChat education module anytime and anywhere. When they had problems, they could consult other experienced parents on the WeChat platform, and they could consult the medical staff at 18:00–22:00 every day. Thus, the parents could more easily and continuously receive professional support through the WeChat platform. Therefore, the WeChat platform improved parents’ understanding of perioperative care knowledge and reduced surgical cancellation. The WeChat platform could increase the medical compliance of patients’ families, increase the understanding of the disease and confidence in treatment, and thus improve the satisfaction of the medical treatment process.

Because of the quite limited medical resources in China, the relatively large number of patients and the limited time and space, most patients and doctors did not have enough time to communicate, which might lead to patients’ dissatisfaction with the medical treatment process. WeChat provided a platform that was not limited to time and space, built a bridge of doctor-patient communication, and had interests to improve doctor-patient communication. Therefore, the scores of general satisfaction, interpersonal manner, communication, time spent with a physician, and accessibility and convenience of the WeChat group were significantly higher than those of the leaflet group. It was free to apply for a WeChat account, and the network fees caused by WeChat operation were almost negligible in China. Besides, medical staff could not charge any fees in this communication process. So using the WeChat platform did not increase the cost of patients.

Compared with the rate of attrition in the control group, the WeChat group was lower. Medical staff could directly contact the children’s parents via the WeChat platform to timely and repeatedly remind patients to follow-up, which could effectively help them follow up on time. This communication method was sustainable, convenient, and inexpensive and was not restricted by regions or time, which could effectively reduce the loss of contact between family members and physicians. However, the patients in the control group only received an education leaflet with a date of follow-up. The parents were prone to ignore the disease and forget to follow up on time because their children had no other symptoms.

There were some limitations to this study. First, due to unstable internet support and demanding access to WeChat platform, the heterogeneity to access the WeChat made difficult the generalizability of these results. Second, this was a single-center study with a relatively small sample size in one region. Studies in other regions, a larger patient population and other parents with different cultural backgrounds and levels of knowledge might yield different results.

## Conclusion

Providing perioperative health education to parents of children who undergo transthoracic device closure of VSDs through the WeChat platform can effectively enhance parents’ knowledge of care, improve parents’ satisfaction, and is an effective method to ensure convenient operation and reduce loss to follow-up.

## Data Availability

Data sharing not applicable to this article as no data sets were generated or analyzed during the current study.

## Abbreviations

VSD: Ventricular septal defect
PSQ-18: Patient Satisfaction Questionnaire-18
